# Impact of non surgical periodontal therapy on glycemic control in individuals with type 2 diabetes mellitus and periodontitis

**DOI:** 10.1590/1678-7757-2025-0121

**Published:** 2025-07-14

**Authors:** Rafael Paschoal ESTEVES LIMA, Karolina Skarlet Silva VIANA, Pierre Geraldo Braz da SILVA, Luís Otávio Miranda COTA, Fernando Oliveira COSTA

**Affiliations:** 1 Universidade Federal de Minas Gerais Faculdade de Odontologia Departamento de Clínica, Patologia e Cirurgias Odontológicas Belo Horizonte MG Brasil Universidade Federal de Minas Gerais, Faculdade de Odontologia, Departamento de Clínica, Patologia e Cirurgias Odontológicas, Belo Horizonte, MG, Brasil.

**Keywords:** Glycemic control, Dental scaling, Diabetes mellitus, type 2, Periodontitis

## Abstract

The infectious and inflammatory process of the periodontal tissues can contribute to hyperglycemia in patients with diabetes.

**Objective:**

The objective of this study was to evaluate the effect of non-surgical periodontal therapy on glycemic control in individuals with type 2 diabetes mellitus.

**Methodology:**

In this clinical trial with two months of follow-up, 31 participants were included, with 15 having adequate glycemic control and 16 inadequate glycemic control. The participants underwent non-surgical periodontal therapy. Biological, social, and behavioral variables were collected. Periodontal clinical examination was performed at baseline and two months after the intervention. Laboratory tests to assess serum levels of glycated hemoglobin, fasting glucose, and C-reactive protein were requested for all participants at baseline and two months after periodontal treatment.

**Results:**

The difference in glycated hemoglobin levels between baseline and two months after non-surgical periodontal therapy was statistically significant in the total sample (p=0.045) and in the group of individuals with adequate glycemic control (p=0.016). No significant difference was observed in glycated hemoglobin levels in the group of individuals with inadequate glycemic control. No significant variation was observed in fasting glucose and C-reactive protein levels after treatment in the studied sample. A reduction in probing depth, gingival inflammation, and gain in clinical attachment was observed in the total sample and in both groups according to glycemic control.

**Conclusion:**

Periodontal intervention may contribute to improved glycemic control in individuals with type 2 diabetes mellitus and periodontitis (Brazilian Clinical Trials Registry RBR-9fvwk4m).

## Introduction

Periodontitis is an inflammatory disease of bacterial etiology prevalent in the global population. The inflammatory response triggered by dysbiosis is the main factor responsible for the damage to the periodontal attachment tissues that characterize this periodontal disease. Clinically, it is primarily characterized by increased probing depth, attachment loss, and the presence of bleeding. Other signs and symptoms may be present, including tooth mobility, furcation involvement, gingival recession, and halitosis.^1-3^ This inflammatory disease of the periodontal tissues significantly impacts masticatory function, aesthetics, and quality of life, and its progression can lead to tooth loss. Risk factors such as diabetes mellitus and smoking contribute to the development, extent, and severity of the disease.^1,4^

Diabetes mellitus encompasses a group of metabolic disorders characterized by hyperglycemia caused by insulin production deficiency and/or insulin resistance. Among them, type 2 diabetes mellitus is the most prevalent and has a high incidence in the population.^5^ A family history of diabetes, obesity, and physical inactivity are risk factors for the development of this hyperglycemic disease. Diabetes mellitus presents a significant rate of mortality and morbidity. Diabetes-related complications, both microvascular and macrovascular in nature, include neuropathy, retinopathy, nephropathy, cardiovascular disease and periphral vascular disease.^6,7^

Immune and inflammatory changes inherent to hyperglycemia demonstrate the complications associated with diabetes, including periodontitis. The deficiency in neutrophil function—an important line of defense in periodontal tissues—combined with the exaggerated production of inflammatory mediators by monocytes and macrophages in response to bacterial aggression, explains the impact of diabetes on periodontal tissues.^8^ On the other hand, periodontitis can contribute to insulin resistance and the consequent deterioration of glycemic control in patients with diabetes. The translocation of bacteria and bacterial products associated with periodontitis, such as lipopolysaccharides, into the bloodstream can induce a systemic inflammatory process, resulting in elevated levels of acute-phase and oxidative stress biomarkers, with repercussions on insulin action. In the presence of a defective or altered immune response, these migrating bacteria may contribute to the initiation and progression of inflammatory diseases. Significant variations in the individual host response to microbial infection, particularly in T-cell and monocyte function, contribute to an excessively intense or hyperreactive inflammatory response. In this process, the ability of oral bacterial species to colonize non-oral surfaces also plays a relevant role.^9,10^ Inflammatory mediators such as interleukin 6 (IL-6), C-reactive protein (CRP), and tumor necrosis factor alpha (TNF-α) appear to be involved in this process, particularly competing for specific insulin binding sites and/or inducing the production of other inflammatory cells.^8,11^

In this regard, it is relevant to analyze the effect of resolving the infectious and inflammatory process associated with periodontitis on the reduction of glycated hemoglobin levels. This clinical trial aimed to evaluate the impact of periodontal treatment on glycemic control in individuals with type 2 diabetes mellitus.

## Methodology

### Study design

This is a clinical trial with a 2-month evaluation period, conducted according to the CONSORT (Consolidated Standards of Reporting Trials) guidelines.^12^ This study was registered in the Brazilian Clinical Trials Registry (ReBEC) under the number RBR-9fvwk4m.

### Participants

The sample consisted of individuals with type 2 diabetes mellitus and stage 2 or stage 3 periodontitis^13^ recruited at the Periodontology Clinic of the School of Dentistry at the Federal University of Minas Gerais, Brazil. Individuals aged 30 years or older, with at least fourteen teeth present and who gave consent to participate in the study were included. The following exclusion criteria were considered: smokers, pregnant or lactating women, individuals who had undergone periodontal treatment in the past six months or received antimicrobial and anti-inflammatory therapy in the past three months, individuals with glycated hemoglobin (HbA1c) levels greater than or equal to 9% and/or fasting blood glucose greater than or equal to 250 mg/dl, and individuals who changed their diabetes treatment medication during the study period. Participants were divided into two groups according to their serum HbA1c levels: adequate glycemic control (HbA1c <7%) and inadequate glycemic control (HbA1c ≥7%).

### Intervention

Participants underwent non-surgical periodontal treatment based on the protocol proposed by Quirynen, et al.^14^ (2000), which involved full-mouth scaling and root planing within 24 hours, divided into two sessions, without the use of chlorhexidine. Ultrasonic instruments and manual Gracey and McCall curettes (Hu-Friedy, USA) were utilized for supra- and subgingival scaling and root planing. The duration of each appointment was recorded. Standardized oral hygiene instructions were provided. Periodontal therapy was performed by two trained researchers (P.G.B.S.J. and K.S.V.).

### Outcomes

The periodontal clinical examination was conducted by two trained and calibrated researchers (P.G.B.S.J. and K.S.V.) to record the parameters of probing depth (PD), clinical attachment level (CAL), and bleeding on probing (BOP) for all present teeth. PD was assessed as a secondary outcome and defined as the distance from the gingival margin to the bottom of the sulcus or pocket. CAL was measured from the cementoenamel junction to the bottom of the sulcus or periodontal pocket. BOP was assessed dichotomously for its presence or absence, considering a standardized evaluation time of 20 seconds after probing, which was performed circumferentially using a North Carolina periodontal probe (UNC-15mm). The parameters were recorded at four sites: buccal, mesial, palatal/lingual, and distal. The tooth was excluded from the analyses in cases in which it was impossible to determine the cementoenamel junction, or when it presented gingival morphological alterations, poorly adapted restorations, or excessive calculus. The clinical periodontal examination was performed at baseline (T0) and 60 days after periodontal treatment (T1).

The assessment of intra- and inter-examiner reliability for the clinical parameters PD and CAL was conducted in a sample of five randomly selected patients, who were re-evaluated one week later. The results demonstrated Kappa coefficients and intraclass correlation coefficients greater than 0.80 for both clinical measures.

Fasting blood glucose, HbA1c, and CRP blood tests were requested from all participants at the same evaluation times, at T0 and T1. HbA1c was assessed as the primary outcome. Additionally, biological and sociodemographic data were collected using a structured questionnaire, which included age, gender, education, family history of diabetes, duration of diabetes, medication use, and history of dental visits. The participants’ weight and height were recorded for the calculation of body mass index (BMI).

### Sample size

The sample size was calculated considering 80% power and a 5% significance level. Assuming a mean difference of 1.0 in HbA1c level and a standard deviation of 1.83,^15^ the total sample size was 32 individuals, which was estimated using the paired t-test, considering the significance level (Z_1−α/2_), the power of the test (Z_1−β_), the standard deviation (δ_d_), and the desired minimum difference (∆).


$$n={\left(\frac{\left(Z_{1-\alpha /2}+Z_{1-\beta }\right)\times \delta_{d}}{\Delta }\right)}^{2}$$

### Statistical analysis

Initially, a descriptive analysis of the total sample and a comparison between the groups were performed regarding the variables of interest. The distribution of the data was tested for normality using the Shapiro-Wilk test. Comparisons between groups were performed using the Mann-Whitney test, the Chi-square test, and the Student’s t-test for independent samples, if applicable. To compare the variables within the same group between T0 and T1, the Wilcoxon test and the paired t-test were used, if applicable. All collected data were put into a database, and analyses were performed using statistical software (SPSS 17.0, Statistical Package for Social Sciences, Windows version, SPSS Inc., Chicago, IL), considering a less than 5% significance probability (p<0.05).

## Results

**Figure 1:**
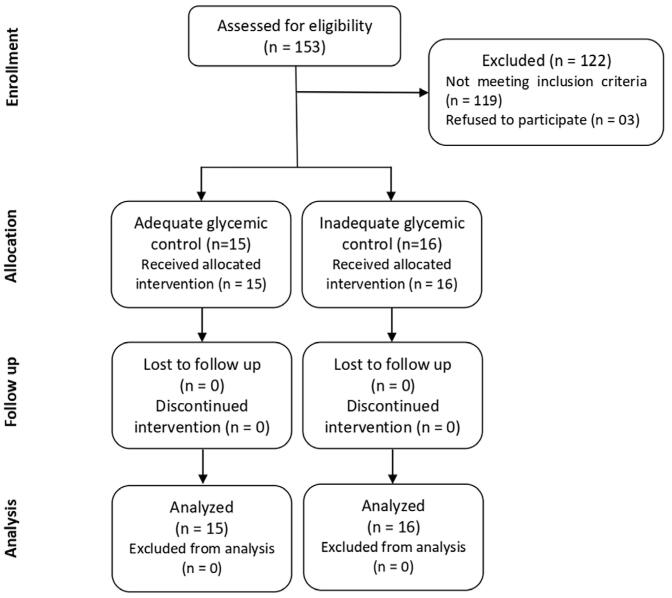
Flowchart of patient inclusion in the study.

A total of 31 participants were included in this clinical trial, with 15 having adequate glycemic control and 16 inadequate glycemic control (Figure 1). The biological and sociodemographic characteristics of the sample are shown in Tables 1 e 2. The sample consisted of individuals with an average age of 58.9 years and an average BMI of 28.4, which indicates they were overweight. The majority of participants had a family history of diabetes. Female individuals were the majority in the group with adequate glycemic control, while the group with inadequate glycemic control had a higher prevalence of male individuals. In addition, individuals with uncontrolled diabetes had an earlier diabetes diagnosis.

**Table 1:** Biological and sociodemographic characterization of the total sample.

Variable	Total sample (n=31)
Age in years (mean±sd)	58.9 (9.7)
Gender n (%)	
Male	14 (45.2)
Female	17 (54.8)
Education n (%)	
<9 years	14 (45.2)
≥9 years	17 (54.8)
Boby mass index mean (mean±sd)	28.4 (3.8)
Duration of diabetes mean (mean±sd)	10.6 (8.1)
Family history of diabetes n (%)	23 (74,2)

**Table 2:** Comparison of biological and sociodemographic characteristics between adequate and inadequate glycemic control groups.

Variable	Adequate glycemic control	Inadequate glycemic control	p
	(n=15)	(n=16)	
Age in years (mean±sd)	60.5 (10.7)	57.4 (8.7)	0.382^†^
Gender n (%)			0.045^‡*^
Male	04 (26.7)	10 (62.6)	
Female	11 (73.3)	06 (37.5)	
Education n (%)			0.376^‡^
<9 years	08 (53.3)	06 (37.5)	
≥9 years	07 (46.7)	10 (62.5)	
Boby mass index mean (mean±sd)	29.0 (4.4)	27.8 (3.1)	0.385^†^
Duration of diabetes mean (mean±sd)	8.33 (8.9)	12.7 (6.8)	0.026^§*^
Family history of diabetes n (%)	10 (66.7)	13 (81.3)	0.354^‡^

^†^t-test, ^‡^chi-square, ^§^Mann-Whitney, p<0.05

The periodontal clinical parameters are described in Tables 3 and 4. There was no difference between the groups for the periodontal parameters assessed at T0 and T1. In both groups, a significant reduction in PD and the percentage of sites with BOP, as well as a significant gain in clinical attachment level, were observed between T0 and T1. The duration of the consultation for periodontal treatment was similar between the groups (p=0.128).

**Table 3:** Periodontal variables in the total sample.

Variable	Total sample (n=31)
Treatment time in minutes (mean±sd)	97.2 (22.0)
Present teeth (mean±sd)	23.2 (5.2)
Sites with bleeding on probing (mean±sd)	
T0	23.2 (18.0)
T1	8.78 (7.2)
p	<0.001^†*^
Probing depth (mean±sd)	
T0	2.64 (0.5)
T1	2.26 (0.4)
p	<0.001^†*^
Clinical attachment level (mean±sd)	
T0	3.26 (0.8)
T1	2.90 (0.8)
p	<0.001^†*^

^†^Wilcoxon, *p<0.05

**Table 4:** Comparison of periodontal variables between adequate and inadequate glycemic control groups.

Variable	Adequate glycemic control	Inadequate glycemic control	p
	(n=15)	(n=16)	
Treatment time in minutes (mean±sd)	105 (21.9)	89.8 (20.0)	0.128^†^
Present teeth (mean±sd)	23.4 (5.2)	23.2 (5.4)	0.912^‡^
Sites with bleeding on probing (mean±sd)			
T0	29.7 (21.3)	17.1 (11.8)	0.105^†^
T1	9.66 (9.2)	7.95 (5.0)	0.890^†^
p	<0.001^§*^	0.003^§*^	
Probing depth (mean±sd)			
T0	2.55 (0.4)	2.72 (0.5)	0.192^†^
T1	2.17 (0.3)	2.34 (0.4)	0.093^†^
p	<0.001^§*^	<0.001^§*^	
Clinical attachment level (mean±sd)			
T0	3.40 (1.1)	3.13 (0.5)	0.953^†^
T1	3.10 (1.1)	2.70 (0.4)	0.384^†^
p	0.009^§*^	<0.001^§*^	

^†^Mann-Whitney, ^‡^t-test, ^§^Wilcoxon, *p<0.05

The laboratory parameters are presented in Tables 5 and 6. Individuals with inadequate glycemic control had higher HbA1c serum levels and fasting glucose at both T0 and T1. There was no difference between the groups regarding serum CRP levels at both evaluation times. A significant reduction in HbA1c levels was observed after periodontal treatment in the overall sample and in the group of individuals with adequate glycemic control. There was no statistically significant difference in HbA1c levels between T0 and T1 among individuals exhibiting inadequate glycemic control. No significant difference in fasting glucose levels was observed between T0 and T1 in either group.

**Table 5:** Biochemical parameters in the total sample.

Variable	Total sample (n=31)
C-reactive protein (mean±sd)	
T0	5.54 (4.0)
T1	4.50 (2.7)
p	0.198^†^
Glycated hemoglobina (mean±sd)	
T0	7.16 (1.0)
T1	7.03 (1.1)
p	0.045^‡*^
Fasting blood glucose (mean±sd)	
T0	142 (51.3)
T1	135 (40.4)
p	0.450^‡^

^†^Wilcoxon, ^‡^paired t-test, *p<0.05

**Table 6:** Comparison of biochemical parameters between adequate and inadequate glycemic control groups.

Variable	Adequate glycemic control	Inadequate glycemic control	p
	(n=15)	(n=16)	
C-reactive protein (mean±sd)			
T0	5.34 (4.4)	5.74 (3.7)	0.536^†^
T1	4.79 (2.7)	4.21 (2.7)	0.673^†^
p	0.919^§^	0.041^§*^	
Glycated hemoglobina (mean±sd)			
T0	6.30 (0.5)	7.97 (0.6)	<0.001^‡*^
T1	6.17 (0.5)	7.84 (0.9)	<0.001^‡*^
p	0.016^||*^	0.275^||^	
Fasting blood glucose (mean±sd)			
T0	117.0 (19.0)	165.0 (61.5)	0.008^‡*^
T1	119.0 (26.3)	151.0 (46.1)	0.029^‡*^
p	0.656^||^	0.375^||^	

^†^Mann-Whitney, ^‡^t-test, §Wilcoxon, ^||^paired t-test, *p<0.05

## Discussion

Genetic and environmental factors can contribute to the reduction of β-cell function or mass, which are responsible for insulin synthesis and secretion, promoting the development of hyperglycemia.^16^ Insulin resistance may be associated with an inflammatory process, which is a primary mechanism related to the pathophysiology of diabetes.^17^ It has been shown that individuals with diabetes mellitus have elevated levels of inflammatory mediators such as TNF-α and CRP.^18,19^ In this context, the infectious and inflammatory process associated with periodontitis could contribute to insulin resistance, impairing glycemic control. This clinical trial evaluated the effect of periodontal therapy on the glycemic control of individuals with type 2 diabetes mellitus.

The results demonstrated an improvement in glycemic parameters following periodontal treatment in the group of individuals with adequate glycemic control, as evidenced by the significant reduction in serum HbA1c levels. Clinical studies do not present a consensus regarding the benefit of periodontal therapy in glycemic control in individuals with type 2 diabetes mellitus, showing divergent outcomes.^20-23^ However, despite the limitations and heterogeneities, systematic reviews have supported that periodontal treatment can contribute to improved glycemic control, with varying percentages of HbA1c level reduction.^24-26^

In contrast to the result observed for HbA1c, periodontal therapy did not contribute to a reduction in fasting glucose levels in this clinical trial. Although both tests are used for the diagnosis of diabetes, it is recommended that the fasting glucose test not be the primary method for outpatient monitoring of individuals with diabetes, but rather be used to supplement information from other tests or to evaluate the accuracy of self-monitoring.^27^ The fasting glucose test measures level of glucose in the bloodstream at a specific point in time, considering a fasting period of 8 to 12 hours, while the HbA1c test is the primary tool for assessing glycemic control as it reflects average blood glucose levels over a period of approximately three months.^5,28^ Furthermore, this test has a high predictive value for complications associated with diabetes mellitus.^28^

The sample analysis, which was subdivided into groups according to glycemic control, demonstrated that periodontal therapy in individuals with adequate glycemic control contributed to a significant reduction in HbA1c levels. In contrast, this result was not observed in the group of individuals with inadequate glycemic control. Several other factors can affect the glycemic control of individuals with type 2 diabetes mellitus, including BMI, therapy, patient adherence to treatment, dietary habits, physical activity behaviors, and sleep quality.^29^ In the sample used in this study, individuals with inadequate glycemic control had a similar BMI to those with adequate glycemic control. However, the incidence of other factors in the first group may contribute to the difficulty in glycemic control and minimize the impact of an isolated intervention, such as the resolution of the infectious and inflammatory process in periodontal tissues resulting from periodontitis. Interestingly, periodontal treatment contributed to a reduction in serum CRP levels in the group with inadequate glycemic control, which is a nonspecific marker of inflammation used to assess the presence of inflammation or infection and to evaluate cardiovascular disease risk. Other studies have shown that periodontal therapy contributes to reducing serum CRP levels.^30-32^

The reduction in HbA1c levels resulting solely from the treatment of periodontitis or another infectious and inflammatory process, combined with the reduction achieved via other interventions such as changes in dietary patterns, can amplify the clinical benefits of this variation in HbA1c levels in the blood, impacting morbidity and mortality rates related to this hyperglycemic disease. It is estimated that a 1% reduction in HbA1c levels is associated with an approximately 21% reduction in the risk of any diabetes-related outcome, a 21% reduction for diabetes-related deaths, a 14% decrease in myocardial infarction, and a 37% drop in microvascular complications.^33^ These data highlight the importance of efforts to identify harmful factors and improve glycemic control in individuals with diabetes.

The periodontal intervention demonstrated effectiveness in improving periodontal parameters in the sample of individuals with type 2 diabetes mellitus, resulting in a significant reduction in probing depth and bleeding, as well as a significant gain in clinical attachment levels. Regardless of the systemic impact, the resulting periodontal health provides benefits for masticatory function, preservation of dental elements, aesthetics, and quality of life.^34^ Uncontrolled diabetes is a risk factor for the worsening of periodontal diseases and greater tooth loss. Therefore, periodontal treatment should be emphasized in the general prevention strategies for susceptible individuals. The immune and inflammatory alterations inherent to hyperglycemia are concerning and make other treatments complex and challenging, including periodontal care. However, other clinical trials support the efficacy of periodontal therapy in individuals with type 2 diabetes mellitus, which aligns with the findings of this clinical trial.^15,32,35^

The short follow-up period is a limitation of this study that should be taken into account. Additionally, conducting subgroup analyses considering differences in the severity and extent of periodontitis should be addressed in future studies. The absence of an inflammatory marker assessment is also a limitation of this clinical study. The evaluation of markers, such as interleukin-1, IL-6, CRP and TNF-α, is essential for investigating the association between periodontitis and diabetes mellitus. Moreover, the absence of an assessment of patient adherence to diabetes treatment represents a limitation that should be duly acknowledged and considered in the interpretation of the findings.

## Conclusion

Improving strategies that aim at better glycemic control in individuals with diabetes is crucial, as complications associated with this hyperglycemic disease are directly related to the degree of glycemic control. This clinical trial demonstrated that periodontal therapy in individuals with type 2 diabetes mellitus provides benefits in glycemic control, contributing to a reduction in HbA1c levels.
